# Behavioral correlates of temporal attention biases during emotional prosody perception

**DOI:** 10.1038/s41598-022-20806-3

**Published:** 2022-10-06

**Authors:** Raphaël Guex, Didier Grandjean, Sascha Frühholz

**Affiliations:** 1 Laboratory for Behavioural Neurology and Imaging of Cognition, Campus Biotech, University of Geneva, Geneva, Switzerland; 2 Pre-Surgical Epilepsy Evaluation Unit, Clinic of Neurology, University Hospital, Geneva, Switzerland; 3 Swiss Center for Affective Sciences, University of Geneva, Geneva, Switzerland; 4 Department of Psychology, University of Geneva, Geneva, Switzerland; 5 Department of Psychology and Educational Sciences, University of Geneva, Geneva, Switzerland; 6 Department of Psychology, University of Zurich, Zurich, Switzerland; 7 Neuroscience Center Zurich, University of Zurich and ETH Zurich, 8057 Zurich, Switzerland; 8 Center for Integrative Human Physiology (ZIHP), University of Zurich, 8057 Zurich, Switzerland

**Keywords:** Human behaviour, Emotion

## Abstract

Emotional prosody perception (EPP) unfolds in time given the intrinsic temporal nature of auditory stimuli, and has been shown to be modulated by spatial attention. Yet, the influence of temporal attention (TA) on EPP remains largely unexplored. TA studies manipulate subject’s motor preparedness according to an upcoming event, with targets to discriminate during short attended trials arriving quickly, and, targets to discriminate during long unattended trials arriving at a later time point. We used here a classic paradigm manipulating TA to investigate its influence on behavioral responses to EPP (n = 100) and we found that TA bias was associated with slower reaction times (RT) for angry but not neutral prosodies and only during short trials. Importantly, TA biases were observed for accuracy measures only for angry voices and especially during short trials, suggesting that neutral stimuli are less subject to TA biases. Importantly, emotional facilitation, with faster RTs for angry voices in comparison to neutral voices, was observed when the stimuli were temporally attended and during short trials, suggesting an influential role of TA during EPP. Together, these results demonstrate for the first time the major influence of TA in RTs and behavioral performance while discriminating emotional prosody.

## Introduction

Current theories of human cognition suggest that auditory perception relies on predictive coding^1,2^, with temporal expectancies enabling preparedness to achieve moment-to-moment discrimination or categorization and swift behavioral responses^3^. Emotional appraisal has also been described as a dynamical process taking place over time and being subject to attentional biases during its initial stage^4^. The precise influence of attention on emotional perception has been intensively investigated and consequently debated in the last two decades^5–7^. Behavioral studies on spatial and temporal attention (TA) have both associated longer reactions times and reduced accuracy for processing unattended stimuli^3^. And while a large effort has been dedicated to investigate the influence of spatial attention on emotion perception both in the visual and auditory domain^7^, behavioral evidence of TA bias during emotional perception remain scarce.

**Figure 1:**
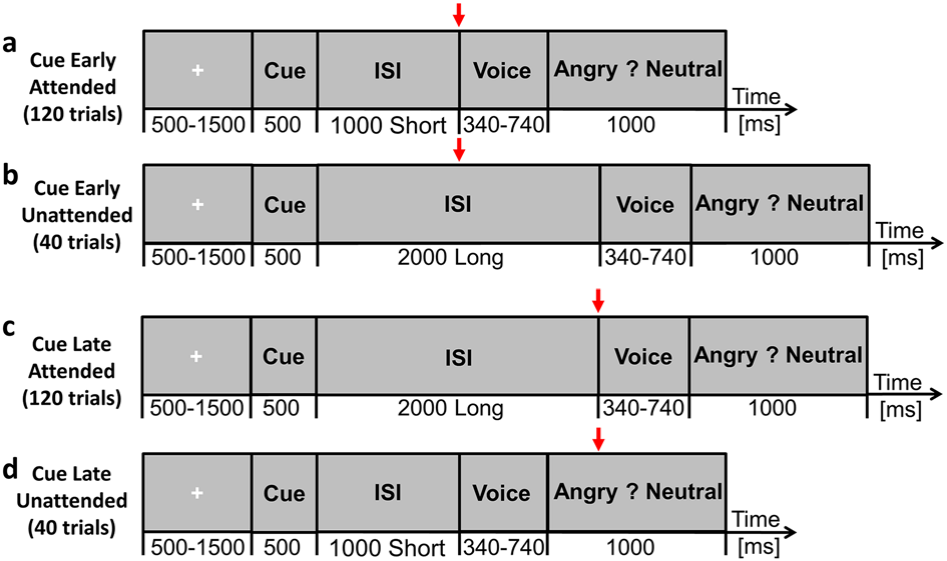
Schematic representation of the emotional recognition task. (**a**) Early attended trials, with short inter-stimulus interval (ISIS). (**b**) Early unattended trials, with long ISIS. (**c**) Late attended trials, with long ISIS. (**d**) Late unattended trials, with short ISIS. Red arrows indicate when the subjects expect the target.

In TA studies, participants have to temporally prepare a motor response based on the information carried by a cue, and consequently attend one stimulus at one specific time point in order to discriminate or categorize it as efficiently as possible. To measure the influence of TA on stimulus processing, the cue information is experimentally manipulated, such that one fraction of the stimuli is temporally attended and the other is not. Usually a ratio of ~ 25% of the cues deliver wrong information to infer the appearance of the target stimulus^3^, enabling the direct comparison between temporally unattended and attended stimuli. While influence of TA has been studied on emotional face perception^8,9^, no study has investigated its influence on EPP. In comparison with visual stimulation, EPP requires temporal integration to assess the emotional significance of a stimulus^10^, inviting further investigation of TA biases on EPP. Recent neurobiological models proposed that the human brain works as temporal predictor during auditory perception, and that top-down control (e.g. TA) may reset the sensory sampling by increasing (or decreasing) the sensory excitability to one stimulus^1^. In this experiment, we used a classic paradigm to investigate if such top-down control of TA might affect EPP. Briefly, the subjects (n = 100) had to categorize the emotional content of a target voice (angry or neutral) as fast and efficiently as possible. To do so, the subjects were given a cue at the beginning of each trial indicated whether the inter-stimulus interval between cue and onset would be short (1 s) or long (2 s; see Fig. 1); in order to evaluate the influence of TA, the cue directed attention to the wrong time in 25% of the trials, leading subjects to expect the target later than it occurred on short trials, thus resulting in an unattended target presentation, and leading subjects to expect the target earlier than it occurred on long trials. This design allows the observation of TA influence over EPP. Based on the literature on emotional appraisal^4^, we hypothesized faster RT for angry in comparison to neutral voices. We further hypothesized that temporally unattended voice targets will be associated to slower response time and lower accuracy during short trials in comparison to temporally attended voice targets, and that such TA biases would be less observable during long trials^3^.

## Results

### Response time

To evaluate first if an emotional vocalization (angry or neutral) could be discriminated differently depending of the cue value (early or late) and the inter-stimulus interval (ISI; short or long), we tested if our three experimental factors (emotion, cue, and ISI) interacted with each other, which was confirmed (*t*(28,679) = 7.56, *p* < 10^–12^, *ES* = 0.73).

To understand what was driving this triple interaction, we next submitted our RTs (response time) to three two-by-two interactions between paired factors, and, we found that each factor significantly interacted with each other (cue x ISI, *t*(28,679) = 6.13, *p* < 10^–7^, *ES* = 0.67, ISI x emotion, *t*(28,679) = 4.76, *p* < 10^–4^, *ES* = 0.47, and cue x emotion, *t*(28,679) = 10.16, *p* < 10^–14^, *ES* = 0.44). We found no main effect of emotion, or cue nor ISI (all *p* > 0.05). To further elucidate these interactions, we next compared each experimental condition for each pairs of factors.

**Figure 2:**
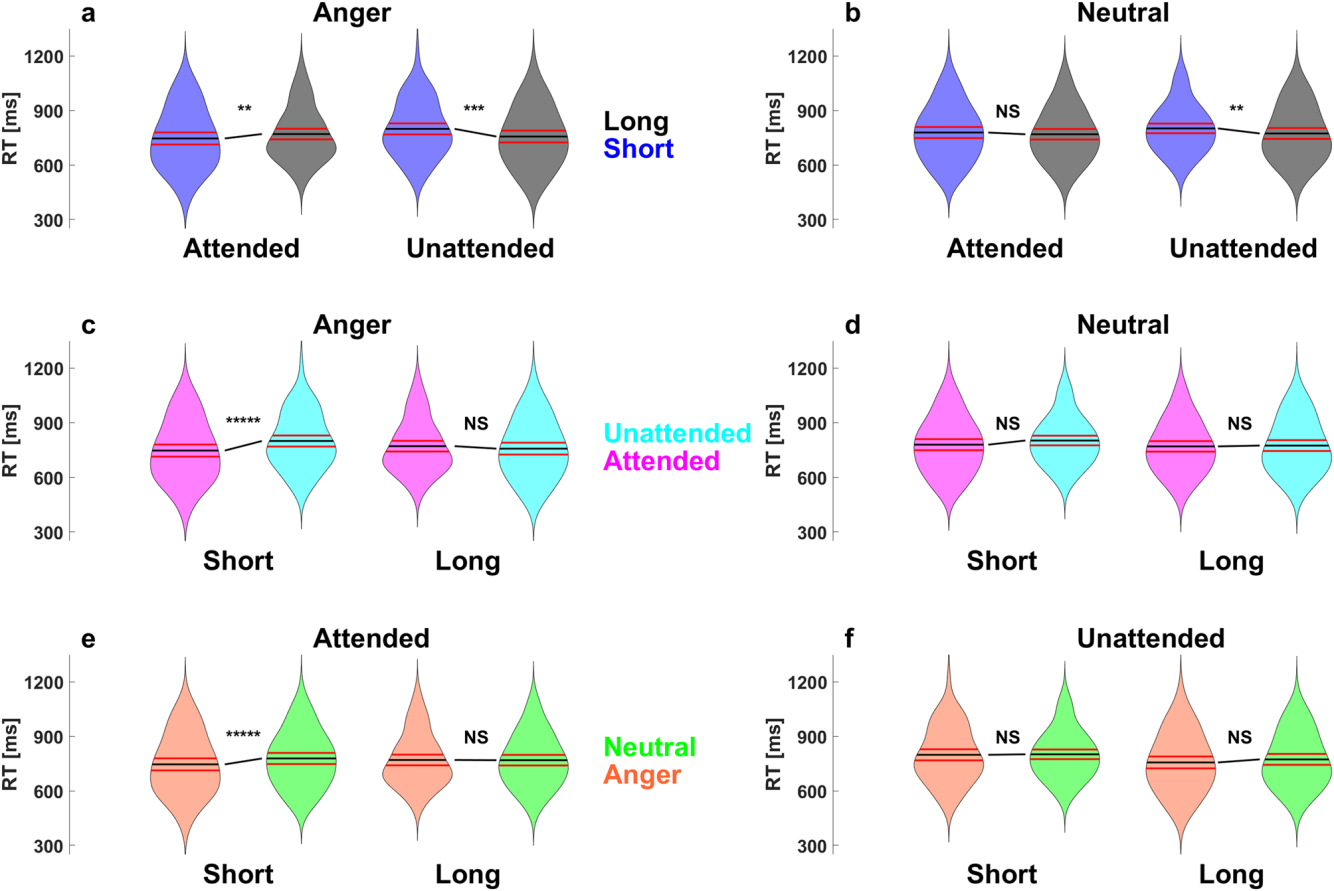
Emotional specific temporal attentional biases in emotional prosody perception in response time. Violin plots of responses time depending on the factors cue, attention and emotion. Cues differences between short (in blue) and long (in grey) trials (Top), for angry voices left and for neutral voices right, depending on the attentional factor (attended and unattended; **a**–**b**). Attentional differences between attended (in magenta) and unattended (in cyan) trials (Middle), for angry voices left and for neutral voices right, depending on the target stimulus onset factor (short and long; **c**–**d**). Emotional differences between angry (in orange) and neutral voices (in green) trials (Bottom), for attended voices left and unattended voices right, depending on the target stimulus onset factor (short and long; **e**–**f**). Note here that the raw results are presented in Table S1. The black line on the violin plot indicate the averaged value and the red lines the Confidence Interval at 95%. The star above this line indicate the statistical significance for each tests, with ***** indicating a Bonferroni corrected *p* value below < 0.00001, **** < 0.0001, *** < 0.001, ** < 0.01, * < 0.05 and NS stands for statistically non-significant*.*

Supporting these interactions, we found first faster RTs during short trials compared to long trial for attended angry voices, (*M*_*short*_ 746 ± 167 ms and *M*_*long*_ 769 ± 146 ms, *z*(99) = − 3.62, *p* = 0.004, *ES* = 0.38) but not for attended neutral voices (*p* > 0.05; Fig. 2a–b). During unattended trials, the opposite pattern was found, as faster RTs were observed during long trials compared to short trial for both angry and neutral voices (respectively, *M*_*short*_ 799 ± 154 ms and *M*_*long*_ 757 ± 165 ms, *z*(99) = 4.11, *p* = 0.0006, *ES* = − 0.45; *M*_*short*_ 801 ± 133 ms, and, *M*_*long*_ 773 ± 151 ms and *z*(99) = 3.9, *p* = 0.001, *ES* = − 0.38; Fig. 2a–b).

Furthermore, as a TA bias we found faster RTs during attended trials compared to unattended trials only for angry voices during short trials (*M*_*attended*_ 746 ± 167 ms and *M*_*unattended*_ 799 ± 154 ms, *z*(99) = − 5.4, *p* = 0.000001, *ES* = 0.62; all other* p* > 0.05; see Fig. 2c–d).

Finally, faster RT for angry voices compared to neutral voices was only found during short and attended trials (*M*_*Anger*_ 746 ± 167 ms and *M*_*neutral*_ 778 ± 153 ms, *z*(99) = − 5.38, *p* = 0.000001, *ES* = 0.59; all other *ps* > 0.05; see Fig. 2e–f).

### Accuracy level

**Figure 3:**
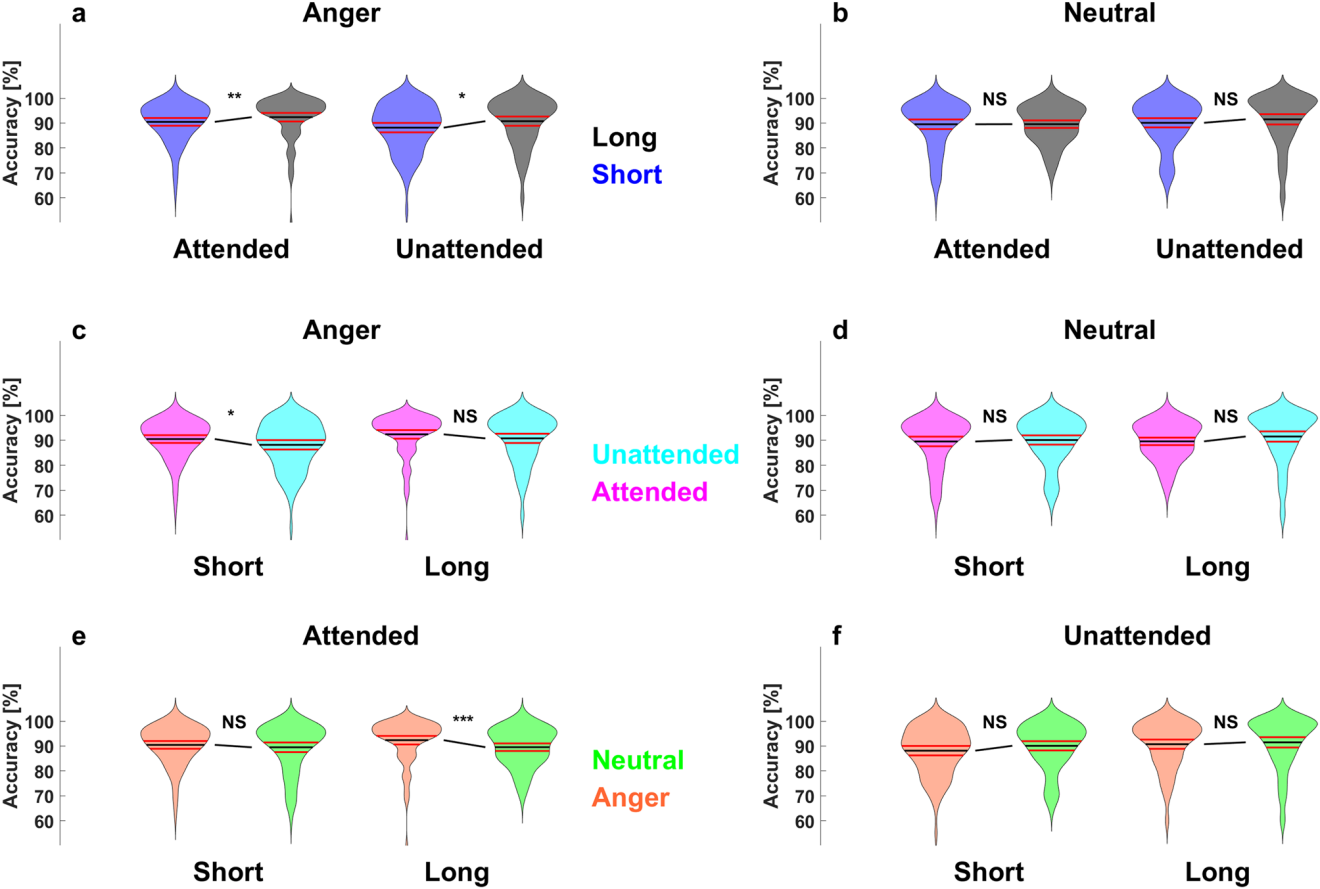
Emotional specific temporal attentional biases in emotional prosody perception in accuracy. Violin plots of accuracy depending on the factors cue, attention and emotion. Cues differences between short (in blue) and long (in grey) trials (Top), for angry voices left and for neutral voices right, depending on the attentional factor (attended and unattended; **a**–**b**). Attentional differences between attended (in magenta) and unattended (in cyan) trials (Middle), for angry voices left and for neutral voices right, depending on the target stimulus onset factor (short and long; **c**–**d**). Emotional differences between angry (in orange) and neutral voices (in green) trials (Bottom), for attended voices left and unattended voices right, depending on the target stimulus onset factor (short and long; **e**–**f**). Note here that the raw results are presented in Table S1. Same color and display codes as in Fig. 2.

Applying the same methodology for analyzing the accuracy, the triple interaction did not reach significance (*p* > 0.05), but significant two-by-two interaction between the factors ISI and emotion (*t*(28,679) = 3.43, *p* = 0.011, *ES* = 0.383), and no main effect (all *ps* > 0.05). Then, again, we compared each experimental condition for each pair of factors (emotion, cue and ISI). Where angry voices were less well recognized during short trials in comparison to long trials during attended and unattended trials (respectively, *M*_*short*_ 90 ± 8% and *M*_*long*_ 92 ± 9%, *z*(99) = − 3.77, *p* = 0.002, *ES* = 0.3; *M*_*short*_ 88 ± 10% and *M*_*long*_ 91 ± 9%, *z*(99) = − 3.05, *p* = 0.035, *ES* = 0.24; Fig. 3a), no differences were observed for neutral voices (all *ps* > 0.05; Fig. 3b). Furthermore, as a TA bias we found that angry voices were less well recognized when they were unattended during short trials (*M*_*attended*_ 90 ± 8% and *M*_*unattended*_ 88 ± 10%, *z*(99) = 2.97, *p* = 0.04, *ES* = − 0.29; Fig. 3c), no differences depending on TA were observed for other conditions (all *ps* > 0.05; Fig. 3c–d). Finally, angry voices were recognized with higher accuracy in comparison to neutral voices during long attended trials (*M*_*angry*_ 92 ± 9% and *M*_*neutral*_ 90 ± 8%, *z*(99) = 4.52, *p* = 0.00001, *ES* = − 0.35; see Fig. 3e), no differences were observed for neutral voices (all *ps* > 0.05; Fig. 3e–f).

### Impact of temporal attention on successive trials

To test if TA could bias behavioral responses in the following trial, we compared the trials which were preceded by unattended trials to trials preceded by attended trials. We found that RTs were significantly slower when the preceding trial was unattended in comparison to attended (*M*_*attended*_ 766 ± 144 ms and *M*_*unattended*_ 774 ± 153 ms, *t*(28,922) = 2.9, *p* = 0.003, *ES* = 0.3). Finally, no intertrial TA bias was observed for the accuracy level (*p* > 0.05).

## Discussion

In this study, we aimed at measuring the impact of TA biases on RT and accuracy in the context of EPP, with angry and neutral voices as behavioral target. We found first an interaction in RTs between the three factors of the experiment, the cue, the emotional content and the ISI; importantly, each factor had an impact on RTs and performance, and interacted with another.

As reported before, in the visual^3^ and in the auditory sensory modality^11^, faster RTs were observed during short trials in comparison to long trials (Fig. 2a–b). Interestingly, while the accuracy was lower for angry voices during short trials in comparison to long trials (Fig. 3a), independently of TA, no differences due to ISI were observed for neutral voices (Fig. 3b), suggesting that ISI may have a greater impact on emotional than neutral voices.

As expected, TA bias was observed only during short trials, and interestingly only for angry voices, with slower RTs associated with unattended trials in comparison to attended (Fig. 2c). This result suggests an effect of surprise linked to TA during short unattended trials, with increased costs associated to earlier auditory stimulus occurrence than predicted by the participant. During long unattended trials however, participants had the possibility to reorient their TA to an upcoming event, decreasing therefore their attentional biases. Moreover, these results suggest that neutral voices are less subject to TA biases, possibly because fast response to angry voices are more relevant for survival. Furthermore, TA’s influence appeared to spread to the following trial, with slower RTs associated with unattended trials in comparison to attended, highlighting again its critical importance in the context of EPP.

TA bias was found for the accuracy level only of angry voices during short trials (Fig. 3c–d). Which also suggests that neutral voices recognition may be less susceptible to TA biases, and therefore support the notion that emotionally irrelevant stimuli may be less subject to attentional manipulation related to its lower recognition relevance linked to survival^7^, at least concerning its lower discriminability than angry voice in the context of TA biases. Indeed, neutral voices were recognized with lower accuracy than angry voices during long attended trials (Fig. 3e).

Furthermore, predictive coding is assumed to play a major role in auditory perception^12^. By showing here that TA is observed in RTs during EPP for angry but not neutral voices, our results suggest that auditory prediction for evolutionary relevant stimulus may be disrupted by temporal expectation manipulation leading to increased behavioral performance during temporal preparedness, as observed with shorter RTs and higher accuracy associated with temporally attended angry voice target. Importantly, despite the fact that these results cannot be generalized to other emotional content, such as fearful or happy emotional prosody, nor to implicit processing of EPP, these results also suggest that behavioral response to angry voices may be subject to attentional manipulation, at least in the context of temporal expectation and preparedness, which complements the view of an automatic processing of emotional signals during condition of inattention^5,7,9^ and sensory unawareness^6,13^.

Remarkably, faster recognition of angry compared to neutral voices was observed especially during early attended trials (Fig. 2e). Most studies investigating behavioral and neuronal responses during EPP have used a temporally predictable target onset in speeded RT procedures. This finding appears therefore of high importance in the study of EPP which evolves in time to achieve behavioral significance, and suggest that temporal preparedness indeed influences emotional facilitation of behavioral response during EPP.

These results provide the first empirical support that TA is of major importance during EPP, influencing both RTs and accuracy. Furthermore, these results support the hypothesis that TA acts as a dynamical process for EPP^14,15^, with specific values of the cue to proactively prepare the sensory processing of a target voice^1^, with emotional specificity, and to guide behavior during the anticipation of an upcoming target voice^3,16^. Moreover, TA seems to impact behavioral performance in an emotional specific fashion, which may help to further develop theoretical models of emotional appraisal. Together, these results suggest that predictive coding models shall integrate both emotional value of the stimulus and TA in order to achieve optimal behavioral prediction.

## Methods

### Stimuli

The stimuli consisted of eight meaningless words (“belam”, “molen”, “nolan”, “loman”, “nodag”, “minad”, “lagod”, “namil”) per emotional condition (angry and neutral) taken from the Geneva Multimodal Emotion Portrayal database, each spoken by eight male and eight female speakers, resulting in one hundred twenty eight stimuli. Each stimulus was presented on average 2.5 times. Their mean duration was 540 ± 200 ms and they were equated for mean sound pressure level and their duration did not differ between emotional conditions (*p* > 0.05). Prior to the study, twenty four subjects (four males, mean age 22.1y ± 3.5) evaluated the stimuli over two emotional dimensions on a Likert scale, namely how angry and how neutral they are perceived. Angry voices were judged as more angry in comparison with neutral voices (two-sided t-test, *t*(23) = 18.16, *p* < 0.001) and neutral voices were judged as more neutral in comparison with angry (two-sided t-test, *t*(23) = 26.17, *p* < 0.001).

### Participants

Participants were students participating in the experiment for receiving university credits. In total, one hundred and two subjects took part in the study. The G*Power software^17^ has been used to estimate the sample size required to achieve a high statistical power, with the effect size d calculated over differences of the mean and standard deviation, a minimum of 57 participants was required with Wilcoxon two-sided signed rank test to achieve an effect size of 0.9. All subjects gave their written informed consent prior to the experiment in accordance with ethical guideline of the University of Geneva, which approved this experimental protocol. All were French speakers, had normal or corrected vision and they reported normal hearing abilities, and were all naïve to the purpose of the study. Data from two participants were excluded due to poor performances at chance level. Of the 100 remaining participants, seventy five were female, seventy one were right-handed, and their average age was 25.2y ± 4.3 (range: 21–37y).

### Experimental procedure setting and trial structure

Participants sat at ~ 50 cm of distance from a computer screen (Dell 1908FP 19″ monitor on a Dell OptiPlex 9010 Intel Inside Core i7 vPro computer) and wore Philips SBC HP250 headphones. They received written instructions to respond as fast and correctly as possible, to determine the emotional content of the target vocalization. Once the instructions were read, any questions regarding the procedure were answered.

On each trial, participants were informed with an auditory cue to orient their TA to one specific time point, this time point could be of 1000 or 2000 ms (see below), in order to swiftly determine if the target vocalization was neutral or angry. First, a fixation cross of varying time (500–1500 ms) was presented in order to avoid preparedness, then a cue was presented to the subject informing about the latency of the arrival of the stimulus, which may be fast (1000 ms post-cue onset) or slow (2000 ms post-cue onset). Consequently, the expectancy between cue and target lasted for 1000 or 2000 ms, such difference of latency between early and late trials is known to enable an appropriate observation of TA influence^3,11^. At the same time of presentation of the target, a response screen was presented for 1000 ms; see Fig. 1 for a visual representation of the trial design. The cue was an auditory tone lasting 500 ms (high for early trials: peak at 1000 Hz, low for late trials: peak at 500 Hz). The expectancy period between the cue and the target was a rhythmic auditory tone at 5 Hz, providing an implicit measure of time prediction and therefore enabling optimal observations of TA biases^18^.

Prior to the experiment, a short training of twelve trials with only valid cues (half early trials) was performed. Then, the experiment took place with the cue being valid (target vocalizations expected at a correct time interval) in 75% of the trials for early and late trials, and with an invalid cue in the remaining 25% of the trials, as well for early and late trials, this ratio of ~ 25/75% is known to optimally modulate temporal expectation in order to measure its influence^3^. A total of 320 trials were performed, with equal probability for the target to be an angry or a neutral vocalization. Responses were recorded locked to the onset of the response screen which coincided with the appearance of the stimuli. The experiment was controlled using the software E-prime (Neurobehavioral Systems, Albany, CA, USA).

### Statistical analyses

The three experimental factors were the cue (early or late), target stimulus onset (short or long), and the emotional prosody (angry or neutral). All response times (RT) exceeding ± 3 standard deviation, separately for each experimental condition, and individually for each participant, were excluded from further analyses (representing 3 ± 2.29% of all trials). The interactions between factors were calculated with a General Linear Mixed Model with participants as random effect; note here that the Matlab function used here (fitglme) produces a t value independently of the type of statistical test. The t-test were calculated with paired Wilcoxon two-sided signed rank. All analyses were performed with Matlab 2018. For the accuracy, a binomial distribution was used. A Bonferroni correction for multiple comparisons was used for each analysis^19^, by considering on one hand all statistical tests (16 in total) performed for the RT and on the other hand all statistical tests (16 in total) performed for the accuracy level, leading to a significance alpha threshold of *p* = 0.0031. We present only the corrected results in the main text and present in supplementary Table S1 all corrected and uncorrected results for the sake of completeness. The effect sizes (ES) were computed with Cohen’s method^20^ and violin plots were used for visualization.

## Supplementary Information

Supplementary Information.

## Data Availability

The datasets generated during and/or analyzed during the current study are available from the corresponding author on reasonable request.

## References

[1] Rimmele JM, Morillon B, Poeppel D, Arnal LH (2018). Proactive sensing of periodic and aperiodic auditory patterns. Trends Cogn. Sci..

[2] Skerritt-Davis B, Elhilali M (2021). Computational framework for investigating predictive processing in auditory perception. J. Neurosci. Methods.

[3] Nobre AC, Van Ede F (2018). Anticipated moments: Temporal structure in attention. Nat. Rev. Neurosci..

[4] Sander D, Grandjean D, Scherer KR (2005). A systems approach to appraisal mechanisms in emotion. Neural Netw..

[5] Grandjean D (2005). The voices of wrath: Brain responses to angry prosody in meaningless speech. Nat. Neurosci..

[6] Pessoa L, Adolphs R (2010). Emotion processing and the amygdala: From a ‘low road’ to ‘many roads’ of evaluating biological significance. Nat. Rev. Neurosci..

[7] Vuilleumier P (2005). How brains beware: Neural mechanisms of emotional attention. Trends Cogn. Sci..

[8] Feuerriegel D, Churches O, Coussens S, Keage HAD (2019). Temporal expectations modulate face image repetition suppression of early stimulus evoked event-related potentials. Neuropsychologia.

[9] Pichon S, Guex R, Vuilleumier P (2016). Influence of temporal expectations on response priming by subliminal faces. PLoS One.

[10] Schirmer A, Adolphs R (2017). Emotion perception from face, voice, and touch: comparisons and convergence. Trends Cogn. Sci..

[11] Faugeras F, Naccache L (2016). Dissociating temporal attention from spatial attention and motor response preparation: A high-density EEG study. Neuroimage.

[12] Heilbron M, Chait M (2018). Great expectations: Is there evidence for predictive coding in auditory cortex?. Neuroscience.

[13] Tamietto M, De Gelder B (2010). Neural bases of the non-conscious perception of emotional signals. Nat. Rev. Neurosci..

[14] Fiebelkorn IC, Kastner S (2019). A rhythmic theory of attention. Trends Cogn. Sci..

[15] Haegens S, Zion Golumbic E (2018). Rhythmic facilitation of sensory processing: A critical review. Neurosci. Biobehav. Rev..

[16] Gädeke JC, Föcker J, Röder B (2013). Is the processing of affective prosody influenced by spatial attention? An ERP study. BMC Neurosci..

[17] Erdfelder E, Faul F, Buchner A, Lang AG (2009). Statistical power analyses using G*Power 3.1: Tests for correlation and regression analyses. Behav. Res. Methods.

[18] Ball F, Michels LE, Thiele C, Noesselt T (2018). The role of multisensory interplay in enabling temporal expectations. Cognition.

[19] Sedgwick P (2012). Multiple significance tests: The Bonferroni correction. BMJ.

[20] Cumming G (2014). The new statistics: Why and how. Psychol. Sci..

