# Supplementary material for: Behavioral correlates of temporal attention biases during emotional prosody perception
Source: Sci Rep. 2022 Oct 6;12:16754. doi: 10.1038/s41598-022-20806-3 (PMC9537340; doi:10.1038/s41598-022-20806-3)
Supplement: Supplementary file 1 — Supplementary Information. [file 41598_2022_20806_MOESM1_ESM.docx]

| **Cuying effect** | **Anger Attended** | | | | | | **Anger Unattended** | | | | | |
| --- | --- | --- | --- | --- | --- | --- | --- | --- | --- | --- | --- | --- |
|  | **Short** | **Long** | **Z value** | ***p****corr* | ***p****unc* | ***ES*** | **Short** | **Long** | **Z value** | ***p****corr* | ***p****unc* | ***ES*** |
| **RT** | 746±167 | 769±146 | -3.62 | **0.004** | **0.0002** | 0.38 | 799±154 | 757±165 | 4.11 | **0.0006** | **0.00003** | -0.45 |
| **ACC** | 90±8 | 92±9 | -3.77 | **0.002** | **0.0001** | 0.3 | 88±10 | 91±9 | -3.05 | **0.035** | **0.002** | 0.24 |
|  |  |  |  |  |  |  |  |  |  |  |  |  |
| **Attentional effect** | **Short Anger** | | | | | | **Long Anger** | | | | | |
|  | **Attended** | **Unattended** | **Z value** | ***p****corr* | ***p****unc* | ***ES*** | **Attended** | **Unattended** | **Z value** | ***p****corr* | ***p****unc* | ***ES*** |
| **RT** | 746±167 | 799±154 | -5.39 | **0.000001** | **0.0000006** | 0.62 | 769±146 | 757±165 | 2.31 | 0.33 | 0.02 | -0.19 |
| **ACC** | 90±8 | 88±10 | 2.97 | **0.04** | **0.002** | -0.29 | 92±9 | 91±9 | 1.98 | 0.75 | 0.04 | -0.16 |
|  |  |  |  |  |  |  |  |  |  |  |  |  |
| **Emotional effect** | **Short Attended** | | | | | | **Long Attended** | | | | | |
|  | **Anger** | **Neutral** | **Z value** | ***p****corr* | ***p****unc* | ***ES*** | **Anger** | **Neutral** | **Z value** | ***p****corr* | ***p****unc* | ***ES*** |
| **RT** | 746±167 | 778±153 | -5.38 | **0.000001** | **0.00000007** | 0.59 | 769±146 | 770±149 | -1 | 1 | 0.31 | 0.03 |
| **ACC** | 90±8 | 90±10 | 0.82 | 1 | 0.4 | -0.12 | 92±9 | 90±8 | 4.52 | **0.00001** | **0.000001** | -0.35 |

| **Cuying effect** | **Neutral Attended** | | | | | | **Neutral Unattended** | | | | | |
| --- | --- | --- | --- | --- | --- | --- | --- | --- | --- | --- | --- | --- |
|  | **Short** | **Long** | **Z value** | ***p****corr* | ***p****unc* | ***ES*** | **Short** | **Long** | **Z value** | ***p****corr* | ***p****unc* | ***ES*** |
| **RT** | 778±153 | 770±149 | 1.54 | 1 | 0.1 | -0.15 | 801±133 | 773±151 | 3.9 | **0.0015** | **0.00009** | -0.38 |
| **ACC** | 90±10 | 90±8 | 0.07 | 1 | 0.94 | 0.006 | 90±9 | 92±10 | -1.83 | 1 | 0.06 | 0.16 |
|  |  |  |  |  |  |  |  |  |  |  |  |  |
| **Attentional effect** | **Short Neutral** | | | | | | **Long Neutral** | | | | | |
|  | **Attended** | **Unattended** | **Z value** | ***p****corr* | ***p****unc* | ***ES*** | **Attended** | **Unattended** | **Z value** | ***p****corr* | ***p****unc* | ***ES*** |
| **RT** | 778±153 | 801±133 | -2.67 | 0.11 | 0.007 | 0.31 | 770±149 | 773±151 | -0.47 | 1 | 0.63 | 0.06 |
| **ACC** | 90±10 | 90±9 | -0.81 | 1 | 0.41 | 0.08 | 90±8 | 92±10 | -2.6 | 0.14 | 0.009 | 0.21 |
|  |  |  |  |  |  |  |  |  |  |  |  |  |
| **Emotional effect** | **Short Unattended** | | | | | | **Long Unattended** | | | | | |
|  | **Anger** | **Neutral** | **Z value** | ***p****corr* | ***p****unc* | ***ES*** | **Anger** | **Neutral** | **Z value** | ***p****corr* | ***p****unc* | ***ES*** |
| **RT** | 799±154 | 801±133 | 0.65 | 1 | 0.51 | -0.02 | 757±165 | 773±151 | -1.76 | 1 | 0.076 | 0.22 |
| **ACC** | 88±10 | 90±9 | -2.02 | 0.67 | 0.04 | 0.18 | 91±9 | 92±10 | -0.98 | 1 | 0.32 | 0.06 |

***Legend Table S1***

***Summary of RT and accuracy simple-effect results with standard deviations.*** *With Z-values, p-value corrected (corr) in bold and uncorrected (unc), and effect size.*
